# Single‐Breathhold 3D MR Elastography in the Liver, With Simultaneous R2* and PDFF Mapping

**DOI:** 10.1002/mrm.70372

**Published:** 2026-04-19

**Authors:** Donovan P. Tripp, Anne‐Sophie van Schelt, Omar Darwish, Karl P. Kunze, Shamsa Al Harthy, Nader S. Metwalli, Ahmed M. Gharib, Claudia Prieto, René M. Botnar, Ralph Sinkus, Radhouene Neji

**Affiliations:** ^1^ Research Department of Imaging Physics and Engineering, School of Biomedical Engineering and Imaging Sciences King's College London London UK; ^2^ MR Application Predevelopment Siemens Healthcare Limited Erlangen Germany; ^3^ MR Research Collaborations Siemens Healthcare Limited Camberley UK; ^4^ National Institute of Diabetes and Digestive and Kidney Diseases Bethesda MD USA; ^5^ School of Engineering Pontificia Universidad Católica de Chile Santiago Chile; ^6^ Millennium Institute for Intelligent Healthcare Engineering Santiago Chile; ^7^ Institute for Biological and Medical Engineering Pontificia Universidad Católica de Chile Santiago Chile; ^8^ INSERM, U1184, LVTS Université Paris Diderot Paris France

**Keywords:** liver, MASLD, MR elastography, PDFF, R2*

## Abstract

**Purpose:**

To develop a sequence for the rapid acquisition of MR elastography (MRE) parameters in 3D, with simultaneous measurement of proton‐density fat fraction (PDFF) and R_2_* for multiparametric assessment of liver disease.

**Methods:**

The proposed sequence uses an interleaved motion‐encoding scheme to acquire 3D volumes of all motion encodings and wave offsets from a single series of readouts with constant repetition time. This leads to a highly time‐efficient acquisition, which together with incoherent k‐space undersampling permits all MRE data to be acquired in a single breathhold. The undersampled volumes are reconstructed using compressed sensing with model‐based temporal regularisation. The sequence can be combined with a multi‐echo readout to allow 3D PDFF and R_2_* maps to be reconstructed from the reference volume of the MRE acquisition. Validation was performed in phantoms and in eight healthy volunteers.

**Results:**

Phantom measurements of all viscoelastic parameters, PDFF, and R_2_* corresponded closely with reference measurements. Average liver tissue parameter values acquired with the combined MRE, PDFF and R_2_* sequence at 3 T were 1.36 ± 0.15 m/s for shear‐wave speed, 1.78 ± 0.41 kPa for the magnitude of the complex shear modulus, 0.70 ± 0.16 kPa for the loss modulus, 3.6% ± 1.0% for PDFF, and 46.9 ± 7.3 s^−1^ for R_2_*.

**Conclusion:**

Single‐breathhold 3D MRE in the liver was achieved with a novel acquisition ordering. The sequence offers 3D elastograms from a 16‐s breathhold, or 3D elastograms with co‐registered PDFF and R_2_* maps from a 21‐s breathhold.

## 1 Introduction

Magnetic Resonance Elastography [1] (MRE) enables non‐invasive estimation of biomechanical tissue properties in vivo [2, 3]. MRE has been successfully applied across various domains, including the pancreas [4, 5], kidney [6, 7, 8], brain [9, 10], and breast [11, 12], but its most promising clinical application is in the liver, where it enables fibrosis to be assessed and staged non‐invasively [13, 14, 15], with comparable accuracy to histopathology [16, 17].

Metabolic dysfunction‐associated steatotic liver disease (MASLD) is a highly prevalent condition, affecting approximately one‐fourth of the global population [18, 19]. MASLD begins with fat infiltration (hepatic steatosis), which may progress to metabolic dysfunction‐associated steatohepatitis (MASH), characterised by histological features including lobular inflammation and hepatocyte ballooning. This triggers the rapid formation of scar tissue (fibrosis), which in the advanced stages can cause cirrhosis and hepatocellular carcinoma [20]. Early diagnosis and precise staging of fibrosis are essential for effective disease management and the prevention of complications [21]. Moreover, differentiating MASH from MASLD is critical, as the rapid fibrosis progression associated with MASH significantly impacts long‐term outcomes and mortality rates. Two‐dimensional MRE offers a means to quantify elastic properties of tissue which correlate with the extent of fibrosis [22, 23], and 3D MRE has additionally demonstrated high accuracy in evaluating liver inflammation [24, 25, 26]. 3D MRE thus presents a means to comprehensively and non‐invasively assess the progression of MASH and late‐stage MASLD.

MRE employs external mechanical oscillations to generate mechanical waves, captured at multiple phases of the periodic motion using motion‐sensitised sequences. Imaging with multiple motion‐encoding directions then allows the 3D wavefield to be reconstructed [2]. From this, various clinically relevant viscoelastic parameters can be derived, including the shear wave speed (c_s_, m/s) and the magnitude of the complex shear modulus (|G*|, kPa), each associated with fibrosis, as well as the loss modulus (G_l_, kPa), which is associated with inflammation [24, 26]. Currently, standard 3D MRE requires acquisition across multiple breathholds due to the need to sample all combinations of multiple wave offsets and motion‐encoding directions. This can be uncomfortable for patients and may result in misalignment between successive acquisitions, which adversely affects the accuracy of 3D MRE inversion. Additionally, existing volumetric 3D MRE techniques typically acquire data in a slice‐by‐slice manner, foregoing the signal‐to‐noise ratio (SNR) benefits of 3D slab‐selective acquisitions (with two phase encoding directions). Different acceleration strategies have been demonstrated for single breathhold MRE in the liver and pancreas, including simultaneous multislice excitation [26] and compressed sensing [27]. However, simultaneous multislice techniques can suffer from a loss of wave quality due to the *g*‐factor‐related SNR penalty [26], and existing attempts to achieve single breathhold imaging using compressed sensing have been forced to rely on very high acceleration factors (up to 15) [27], which compromise the obtained image quality.

While MRE can assess the inflammation and fibrosis associated with MASH and late‐stage MASLD, another essential component of a comprehensive MRI liver exam for suspected MASLD patients is the assessment of fat [28] and iron [29] content in the liver. Fat infiltration drives the progression of MASLD, and the early identification of increased liver fat can allow for intervention before irreversible damage is done. The extent of fat deposition in the liver can be quantified through measurement of the proton‐density fat fraction (PDFF) using chemical‐shift encoded (CSE) techniques [30, 31, 32, 33, 34, 35] which have proven highly accurate in classifying steatosis grades [36]. Many MASLD patients also present with iron overload [37], which can be detected through elevated R_2_* relaxation [38]. Liver R_2_* and PDFF maps can be acquired simultaneously in a single breathhold [39], but this would typically be performed separately to any breathholds for MRE acquisition, which may lead to misalignment between different quantitative datasets, which complicates the analysis. This also further increases the total number of breathholds for patients, which may lead to patient fatigue.

To address these limitations, we propose a 3D slab‐selective, rapid spoiled gradient‐echo sequence with optimised timing and incoherent undersampling, paired with an iterative reconstruction scheme for single breath‐hold liver MRE. Furthermore, we propose an extension of this sequence using a multi‐echo bipolar readout, designed to enable simultaneous mapping of MRE‐derived viscoelastic parameters, PDFF, and R_2_* in the liver within a single breathhold.

## 2 Methods

### 2.1 Sequence Design

#### 2.1.1 3D Slab‐Selective MRE

The design of the proposed 3D MRE sequence is based on a slab‐selective phase‐contrast spoiled gradient‐echo acquisition, beginning with a fast, low‐resolution 3D FLASH [40] acquisition for the estimation of coil sensitivities, followed immediately by the commencement of the mechanical vibration and the MRE acquisition within the same breathhold.

**FIGURE 1:**
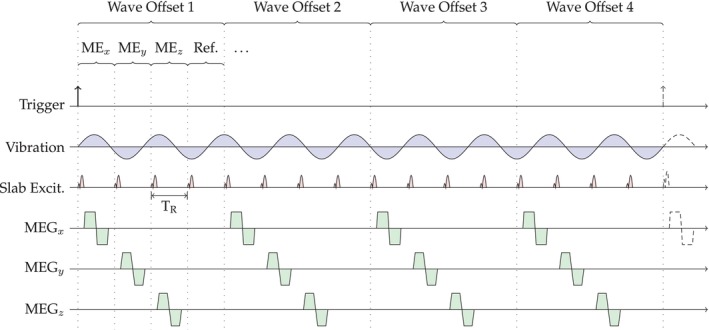
Sequence diagram for the acquisition of one k‐space point in the 2D phase‐encoding plane. The sequence computes the repetition time such that after the consecutive acquisition of three motion encodings, plus the reference without motion encoding, the acquisition is shifted to the next wave offset. Similarly, after the acquisition of four wave offsets, the acquisition has shifted back to the first wave offset and the acquisition of the next phase‐encoding point can proceed immediately. Abbreviations: ME, motion encoding; MEG, motion‐encoding gradient; slab excit., slab excitation.

A schematic diagram of the proposed MRE acquisition scheme is given in Figure 1. The inner loop of the MRE acquisition follows an unbalanced four‐point motion‐encoding scheme [41], consisting of three motion encodings in orthogonal directions, and a reference scan without motion encoding. The sequence implementation computes the repetition time (T_R_) that satisfies the following condition: after the consecutive acquisition of three motion encodings plus reference, the next T_R_ falls exactly at the next wave offset, avoiding the need for a separate time delay, in a manner similar to that of the Ristretto MRE sequence [42]. This is repeated until all wave offsets are acquired, at which point the acquisition of the next k‐space point in the 2D phase‐encoding plane can proceed in the same fashion. A trigger pulse at the commencement of the acquisition of each phase encoding maintains synchronisation with the mechanical vibration.

Through this design, the proposed sequence allows the measurement of all wave offsets and motion encodings for a 3D slab in a single measurement with constant repetition time, leading to a very time‐efficient acquisition. Furthermore, the design of the sequence guarantees that, for each motion‐encoding direction, all locations in the phase‐encoding plane are acquired at the same wave motion state, thereby avoiding motion‐related ghosting artefacts.

**FIGURE 2:**
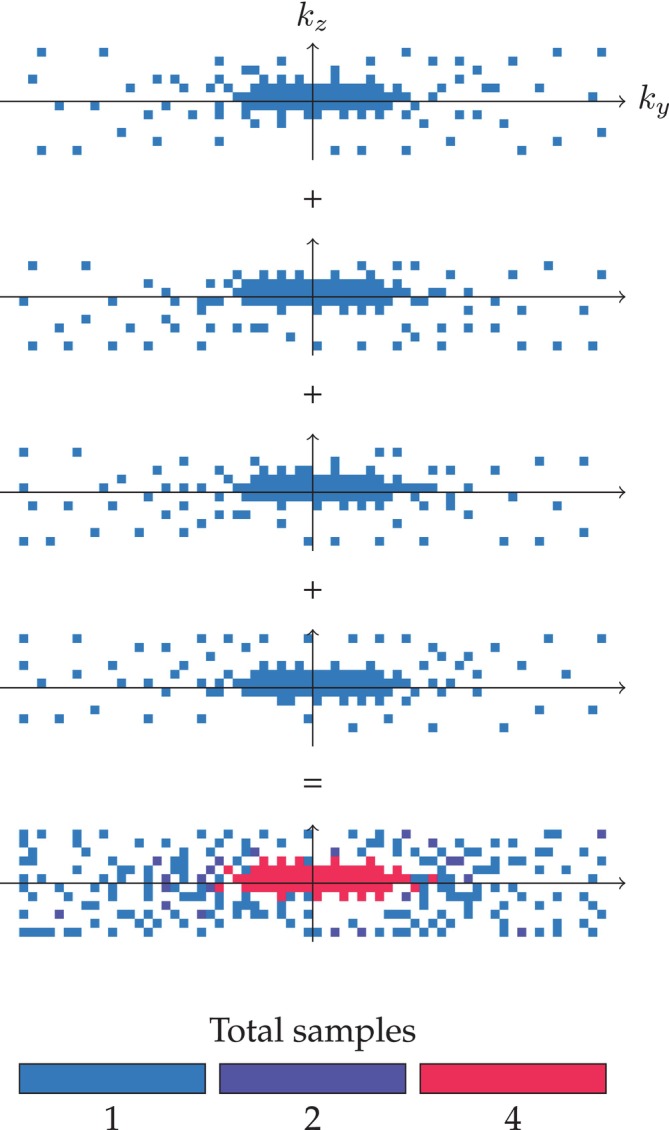
Four incoherent 2D phase‐encoding Poisson‐disc sampling masks used for the different wave offsets (top), and the total k‐space coverage that is obtained through their combination (bottom), which is used for PDFF and R_2_* mapping.

The acquisition is accelerated using a variable‐density Poisson‐disk undersampling pattern that fully samples the central 20% of k space and undersamples the k‐space periphery. To introduce temporal incoherence between the acquired volumes of each motion encoding, and improve overall k‐space coverage, different sampling masks are used for each wave offset, examples of which are shown in Figure 2. The sampling mask is kept constant for the different motion‐encoding directions of a given wave offset.

#### 2.1.2 Integration of PDFF and R_2_* Mapping

**FIGURE 3:**
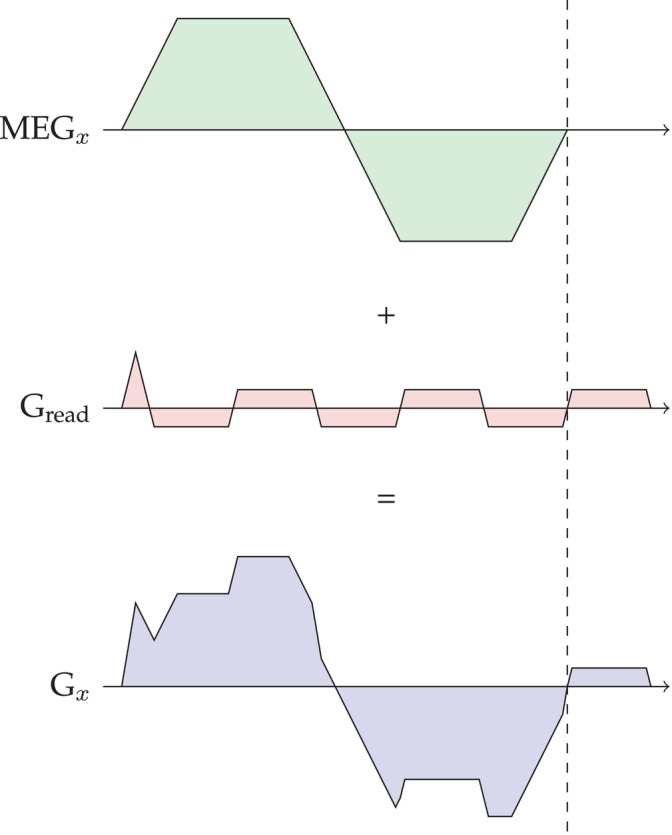
Illustration of the gradient waveform that results from the superposition of the six‐lobe bipolar readout with the bipolar motion‐encoding gradient, whenever the motion encoding is in the readout direction. The first five lobes produce no echo due to the spoiling of the motion‐encoding gradient, but the gradients are timed such that the sixth lobe is unaffected. For other motion‐encoding directions, there is an identical overlap in the timing of the motion‐encoding and readout gradients, although they occur on different gradient axes.

To integrate PDFF and R_2_* mapping into the proposed sequence, a six‐echo bipolar readout [30] can be included within the MRE acquisition. The expected increase in T_R_ can be avoided by overlapping the timing of the first five lobes of the bipolar readout gradient with that of the motion‐encoding gradients, leading to the readout gradient waveform being superimposed onto that of the motion‐encoding gradient whenever the motion encoding is in the readout direction, as depicted in Figure 3. This approach allows the addition of PDFF and R_2_* mapping to be achieved without any increase in repetition time, but entails that the first five lobes of the readout gradient do not produce an echo in the motion‐encoded acquisitions. On the other hand, the reference scan without motion encoding produces six echoes that can be used for PDFF and R_2_* estimation. Only the echo produced by the final lobe of the readout gradient from each motion‐encoded and reference volume is used for MRE quantification. Nonetheless, it is crucial that the full six‐lobe readout gradient is included in the acquisition of all volumes to ensure that the motion‐encoded acquisitions each exhibit the same background phase as the reference volume.

### 2.2 Image Reconstruction

#### 2.2.1 MRE

This work uses a compressed‐sensing [43] scheme for the reconstruction of undersampled data, exploiting redundant information between the different wave offsets acquired for each motion encoding. Two regularising assumptions were employed: (1) that the magnitude of the acquired signal can be considered not to vary between wave offsets, and (2) that variations in the signal phase between wave offsets are dominated by the fundamental temporal frequency component, which corresponds to the mechanical frequency. Under these assumptions, the complex MR signal for a given motion encoding at time *t* and position *r* can be idealised as

(1) $$\hat{x}(r,t)=m(r)\exp(j[p(r)+a(r)\cos(\omega_{\text{mech}}t+\phi (r)]),$$

where *m*, *p*, *a*, and ϕ are spatially varying parameters to be determined: *m* represents the magnitude of the MR signal, *p* the background phase, and *a* and ϕ parameterise the sinusoidal phase accrual due to encoding of the mechanical vibrations at the frequency ω_mech_.

If these parameters were known, then this signal model could be used as a prior in a Tikhonov‐regularised [44] SENSE [45] reconstruction, with the objective function

(2) $$||y-Ex|{|}_{2}^{2}+\lambda ||x-\hat{x}|{|}_{2}^{2},$$

where *y* is the measured data, *x* the reconstructed images, and λ a regularisation weight. The SENSE encoding operator *E* includes the coil sensitivities estimated from the acquired low‐resolution FLASH scan, as well as the Fourier and sampling operators. While the parameters of $\hat{x}$ are not known a priori, this regularised reconstruction can still be realised by estimating them iteratively over the course of the reconstruction, using a version of the Gauss‐Seidel method. This algorithm seeks to reduce the optimisation cost through alternate adjustments to either *x* or $\hat{x}$. Minimising with respect to *x* while taking $\hat{x}$ to be fixed recovers the original Tikhonov‐regularised problem, and so these iterations closely resemble those of a standard gradient‐descent implementation of iterative SENSE [46], but with an additional term in the update of the gradient to reflect the fact that the value of $\hat{x}$ may have changed between iterations. Conversely, by taking *x* to be fixed, the cost can be minimised with respect to $\hat{x}$ by fitting its parameters to the current value of *x*. The time‐invariant magnitude image *m* is taken to be the magnitude of *x* averaged over the wave offsets, and similarly the background phase *p* is the average phase of *x*. The remaining parameters, *a* and ϕ, are taken respectively from the magnitude and phase of the first harmonic in the temporal Fourier representation of the phase of *x*. To achieve accurate estimation of the Fourier components of the phase, 3D spatial phase unwrapping was applied in each iteration using a quality‐map‐based algorithm [47].

Except where stated otherwise, for all experiments, a fixed iteration count of N_iter_ = 100 was used, with a regularisation weight λ = 0.3.

#### 2.2.2 PDFF and R_2_*

PDFF and R_2_* quantification require reconstruction of all the echoes of the reference volumes. Unlike the MRE reconstruction problem, any signal variation between wave offsets is not of interest here, so only the temporal average of the signal across wave offsets need be considered, and hence the k‐space data from all wave offsets can be combined into a single k space for each echo prior to reconstruction. Specifically, as the different wave offsets were acquired with incoherent undersampling, this was achieved by placing the data directly into a single k‐space, except at the fully sampled k‐space centre, where the data from different wave offsets was averaged. This process provides a sufficient reduction in the degree of undersampling for the combined volumes to be reconstructed using iterative SENSE [46] without regularisation.

### 2.3 Quantification of Tissue Parameters

#### 2.3.1 MRE

The sequence design ensures the correct signal phase relation between the volumes acquired at different wave offsets for a given motion encoding, but there exists an undesirable phase shift between the different motion encodings, including the reference scan. Without correcting for this, it would not be possible to remove the background phase from each motion‐encoded volume by direct subtraction of the reference phase.

The additional phase accumulated by the *n*th motion encoding can be derived by first considering the number of cycles that the mechanical vibration completes in the time between this motion encoding and the reference. As different motion encodings are acquired in consecutive acquisitions, the elapsed time between the *n*th motion encoding and the reference is simply *n*T_R_. In this time, the mechanical vibration has advanced by *n*T_R_/T_vib_ cycles, where T_vib_ is the time period of the mechanical vibration. The accumulated phase is therefore

(3) $$\psi_{n}=2\pi \frac{nT_{R}}{T_{\text{vib}}}.$$

An alternative form can be derived by considering the duration of the complete acquisition of a phase‐encoding point. The sequence guarantees that in this time, the mechanical vibration will undergo an integral number of periods N_vib_. Letting N_WO_ and N_ME_ be respectively the total number of acquired wave offsets and motion encodings including reference (in this work, N_WO_ = N_ME_ = 4 and N_vib_ = 9), then by the definition of N_vib_,

(4) $$N_{\text{vib}}T_{\text{vib}}=N_{\text{WO}}N_{\text{ME}}T_{R}\;\Rightarrow \;\frac{T_{R}}{T_{\text{vib}}}=\frac{N_{\text{vib}}}{N_{\text{WO}}N_{\text{ME}}}$$

Finally, substitution of Equation (4) into Equation (3) gives

(5) $$\psi_{n}=2\pi \frac{N_{\text{vib}}}{N_{\text{WO}}N_{\text{ME}}}n.$$

Therefore this phase must be subtracted from the motion‐encoded volumes prior to quantification of the viscoelastic parameters.

Viscoelastic parameters were determined through a direct inversion method. First, the MRE phase images were unwrapped using a minimum‐cost‐flow approach [48], before a pixel‐wise Fourier transform was performed to obtain the displacement field. Each component of the complex‐valued displacement vector was filtered using a 3D Gaussian kernel of width σ = 0.75 pixels with 3×3×3 pixels support. The inversion is performed by applying the curl operator on the displacement data to eliminate the compressional wave component [49]. Solving the Helmholtz equation using a least‐squares fit on these displacement data results in maps of the shear‐wave speed (SWS), the magnitude of the complex shear modulus (|G*|), and the loss modulus (G_l_).

#### 2.3.2 PDFF and R_2_*

PDFF and R_2_* values were jointly estimated from the six echoes of the combined reference volumes, using a variation of the multi‐step fitting approach proposed by Zhong et al. [35]. An initial estimate for the magnitude of the water and fat signals was obtained from B0‐NICE [50] water‐fat separation applied to the first two echoes. These values were then used to initialise a non‐linear least‐squares curve fitting of the magnitude of the signal equation to the magnitude of the measured echoes, also including a single effective R_2_* parameter which was initialised to a fixed value of 30 s^−1^. A six‐peak fat model [51] was used for fitting of in vivo data, whereas for phantom data a ten‐peak model [52] that better reflects the composition of the phantom was used. The numerical optimisation was performed using the implementation of the Levenberg‐Marquardt algorithm in MATLAB (The MathWorks Inc., Natick, MA, USA).

### 2.4 Experiments

#### 2.4.1 Phantoms

The accuracy of MRE measurements with the proposed technique was validated in two phantoms, one containing ultrasound gel, and the other gelatin prepared for this study following recipe G8 of Elisei et al. (80 g gelatin and 125 g sugar in 1 L water) [53]. All MRE data was acquired using a gravitational transducer [54, 55] at a mechanical frequency of 60 Hz, with four wave offsets and an unbalanced four‐point motion‐encoding scheme [41] (three motion‐encoding directions and a reference phase without motion encoding). Images were acquired at 3 T (MAGNETOM Vida, Siemens Healthineers AG, Forchheim, Germany) using an 18‐channel chest coil and a 32‐channel spine coil. Two versions of the proposed sequence were tested: single‐echo, for elastography only (MRE‐only), and six‐echo, for combined elastography, PDFF, and R_2_* mapping (MRE+PDFF). Matching acquisition parameters included field of view (FOV) = 264 mm × 384 mm × 32 mm, voxel size = 4 mm isotropic, T_E_ = {1.1, 2.2, 3.3, 4.4, 5.5, 6.6} ms, T_R_ = 9.38 ms, 10° flip angle, bandwidth = 1000 Hz/pixel, and a motion‐encoding‐gradient amplitude of 20 mT/m. The only differing acquisition parameters were the acceleration factors of 10 for MRE‐only and 7 for MRE+PDFF. The resulting acquisition durations were 16 s for MRE‐only and 21 s for MRE+PDFF. A standard Ristretto MRE sequence [42] was also acquired to provide MRE reference measurements, with relevant acquisition parameters including FOV = 264 mm × 384 mm × 32 mm, resolution = 4 mm isotropic, T_E_ = 7.38 ms, T_R_ = 95.91 ms, 25° flip angle, bandwidth = 801 Hz/pixel. For the gelatin phantom only, the iteration count of the iterative reconstruction described in 2.2.1 was reduced to N_iter_ = 10. All offline reconstructions were performed on a laptop PC (AMD Ryzen 9 6900HX @ 3.30 GHz, 32 GB RAM).

The performance of PDFF and R_2_* mapping with the MRE+PDFF sequence was assessed separately, in a phantom containing fifteen vials submerged in a water bath (Calimetrix LLC, Madison, WI, USA): eight vials exhibiting a range of PDFF values (0%–100%) and seven vials a range of R_2_* values (40–1500 s^−1^). Acquisition parameters were the same as for the MRE phantoms, except that for these experiments the flip angle was 4°. Reference values were taken from the manufacturer's datasheet. Additionally, parameter maps were obtained from the vendor implementation of a multi‐echo Dixon acquisition (qDixon VIBE), with relevant acquisition parameters including resolution = 1.19 × 1.19 × 3.5 mm, T_E_ = {1.05, 2.46, 3.69, 4.92, 6.15, 7.38} ms, T_R_ = 9 ms, 4° flip angle, bandwidth = 1078 Hz/pixel. For these experiments, PDFF and R_2_* maps were reconstructed from the qDixon VIBE data using the same reconstruction as for the proposed sequence, to allow the ten‐peak fat model to be applied consistently.

#### 2.4.2 In Vivo

In vivo feasibility of both the MRE‐only and MRE+PDFF sequences was validated in a cohort of eight healthy volunteers, with reference data acquired using Ristretto MRE and qDixon VIBE. Participant inclusion criteria were age ≥ 16 years, and exclusion criteria were contraindications to MRI. All acquisition parameters were identical to the phantom MRE experiments, except for the motion‐encoding‐gradient amplitude which was 50 mT/m for MRE‐only and 35 mT/m for MRE + PDFF. Reference PDFF and R_2_* values were taken from the vendor inline reconstruction. Written informed consent was given by all participants, and prior approval for all experiments was given by the institutional Research Ethics Panel.

#### 2.4.3 Repeatability Studies

Repeatability of the proposed approach was also assessed in phantoms and in vivo. In phantoms, ten repetitions of the all relevant sequences under consideration were performed. In vivo repeatability was assessed through a test‐retest study of six healthy volunteers. Each subject underwent two consecutive MRI examinations, having been taken out of the scanner and repositioned prior to the second examination.

### 2.5 Analysis

#### 2.5.1 MRE

Regions of interest (ROIs) were drawn over the liver using the magnitude images of the reference scan of the Ristretto sequence. Single ROIs were drawn in the central four slices, while avoiding tissue boundaries, major vessels and regions of low wave penetration [26]. Additional single ROIs were drawn in the kidneys and the spleen, when present in the FOV, in order to evaluate feasibility of the proposed sequence in tissues exhibiting higher stiffness than normal liver tissue.

The average viscoelastic parameters were taken to be the median over the ROI. The parameters analysed were: shear‐wave speed (SWS, m/s), magnitude of the complex shear modulus (|G*|, kPa), and loss modulus (G_l_, kPa). For in vivo data, the average values for each parameter and each method were compared using repeated measures ANOVA and a post‐hoc multiple comparison with Bonferroni correction. Significance was set at *p* < 0.05. All statistical analyses were conducted using SPSS (version 29.0.2.0 (20), IBM SPSS Statistics, Chicago, USA). No statistical analysis was performed for the phantom data as there was only a single measurement for each technique.

#### 2.5.2 PDFF and R_2_*

For phantom measurements, averages within single ROIs placed centrally in each vial on one slice were calculated. The two vials with the greatest R_2_* values (approximately 900 s^−1^ and 1500 s^−1^ as per the manufacturer) were omitted from analysis.

In vivo, single ROIs were drawn over the entire axial cross‐section of the liver. Single ROIs were additionally placed in the kidneys and spleen, when present in the FOV, to assess feasibility over a wider range of R_2_* values than typically observed in normal liver tissue. Values were then averaged within these ROIs over the whole eight‐slice volume of the proposed MRE+PDFF technique. To account for the slight difference in slice thickness between the acquisitions, values from the qDixon VIBE data were averaged over nine axial slices covering the same axial extent as the MRE+PDFF data.

For all experiments, biases between the proposed and reference measurements were assessed with Bland‐Altman difference analysis [56], including 95% limits of agreement (±1.96 SD), and 95% confidence intervals [57] for each statistic. Paired‐sample *t*‐tests were also performed to establish the significance of these biases, with *p* < 0.05 taken to indicate statistical significance. For phantom experiments, linear regression was also performed between measurements and reference values, with the strength of correlation assessed by the coefficient of determination (adjusted r ^2^).

#### 2.5.3 Repeatability

Repeatability in phantoms was assessed by taking the coefficient of variation of the mean value within the ROI across repeat scans. For in vivo repeatability, intraclass correlation coefficients (ICCs) were computed from the test‐retest data across the six subjects of the repeatability study. ICCs were calculated for absolute agreement assuming a two‐way, mixed effects model, using SPSS for MRE data and MATLAB for PDFF and R_2_* data [58].

## 3 Results

### 3.1 Phantom Experiments

#### 3.1.1 MRE

Median viscoelastic parameters obtained within the ultrasound‐gel and gelatin phantoms are summarised in Table 1. Compared with the Ristretto MRE measurements, in the ultrasound‐gel phantom, MRE‐only values exhibited biases of 0.011 m/s for SWS, 0.015 kPa for |G*|, and less than 0.001 kPa for G_l_, whereas for MRE+PDFF these were 0.010 m/s, 0.013 kPa, and 0.007 kPa respectively. In the gelatin phantom, for MRE‐only these biases were −0.17 m/s for SWS, −0.81 kPa for |G*|, and 0.27 kPa for G_l_; for MRE+PDFF these were 0.05 m/s, 0.20 kPa, and 0.69 kPa, respectively. Results from the repeatability study are given in Supporting Information Table S1. Coefficients of variation across shear‐wave‐speed measurements in the ultrasound‐gel phantom were 0.7% for MRE‐only, 1.1% for MRE+PDFF, and 1.3% for Ristretto, and for the gelatin phantom these were 3.6%, 2.6%, and 2.9% respectively.

**TABLE 1:** Median viscoelastic parameters obtained within ultrasound‐gel (a), and gelatin (b) phantoms, for the two proposed sequences and the Ristretto MRE reference.

	(a) Ultrasound gel		
	MRE‐only	MRE+PDFF	Ristretto
SWS (m/s)	0.905	0.904	0.894
|G*| (kPa)	0.803	0.801	0.788
G_l_ (kPa)	0.160	0.167	0.160

	(b) Gelatin		
	MRE‐only	MRE+PDFF	Ristretto
SWS (m/s)	2.64	2.86	2.81
|G*| (kPa)	6.94	7.95	7.75
G_l_ (kPa)	1.34	1.76	1.07

#### 3.1.2 PDFF and R_2_*

**FIGURE 4:**
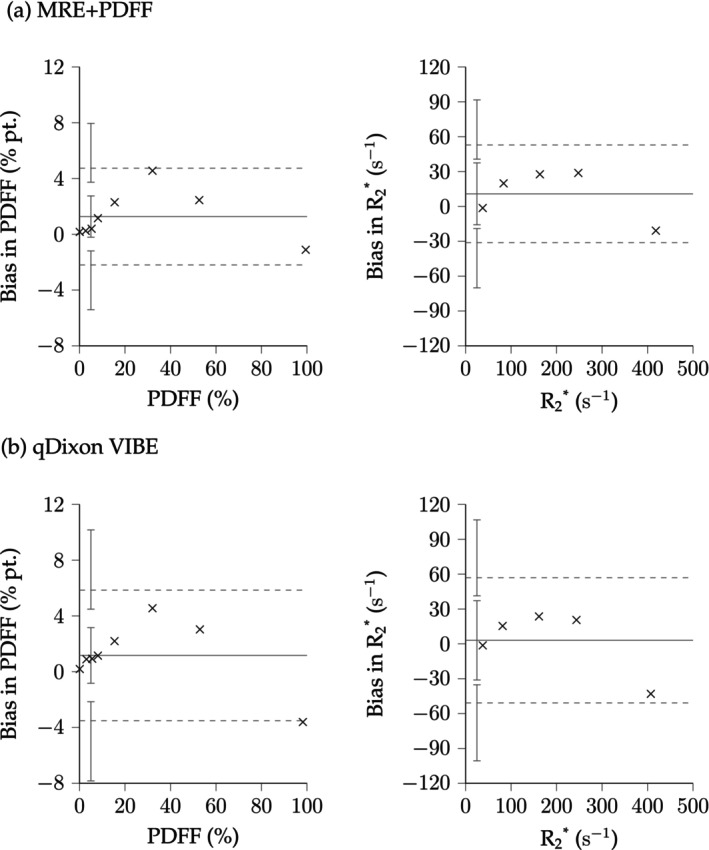
Average per‐vial PDFF (left) and R_2_* (right) values in the PDFF/R_2_* phantom, for the proposed MRE+PDFF (a) and qDixon VIBE (b) sequences compared with reference values provided by the manufacturer. Solid lines denote mean bias, dashed lines give limits of agreement (±1.96 SD) and error bars give 95% confidence intervals of the corresponding statistic.

Figure 4 compares mean per‐vial PDFF and R_2_* measurements from the proposed MRE + PDFF technique and qDixon VIBE with reference values. PDFF values exhibited mean biases of 1.27 percentage points for MRE + PDFF and 1.17 percentage points for qDixon VIBE, and the mean biases in R_2_* values were 10.8 s^−1^ and 3.1 s^−1^ for the proposed technique and qDixon VIBE respectively. None of these biases were statistically significant. 95% limits of agreement for PDFF were {−2.2, 4.7}% for the proposed technique and {−3.5, 5.6}% for qDixon VIBE, and for R_2_* these were {−31.2, 52.9} s^−1^ and {−50.8, 56.9} s^−1^ respectively. Results from linear regression analysis can be found in Supporting Information Figure S1, and results from the repeatability study are given in Supporting Information Figure S2.

### 3.2 In Vivo Experiments

#### 3.2.1 MRE

**FIGURE 5:**
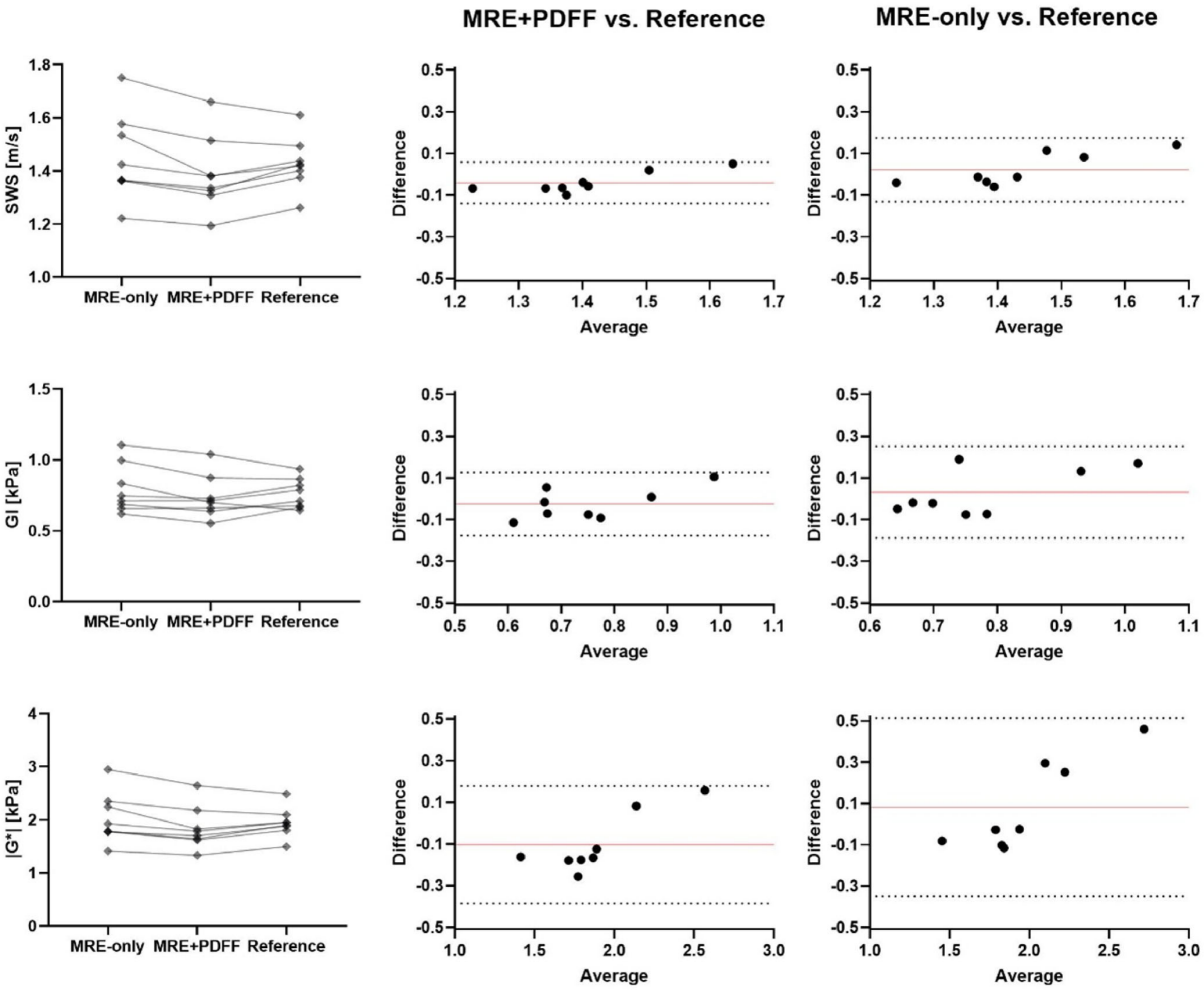
(Left) the average SWS, G_l_, and |G*| for MRE‐only, MRE + PDFF, and the four‐breathhold Ristretto MRE acquisition in the liver for each subject. There is a slight difference in average SWS between MRE+PDFF and Ristretto MRE (p = 0.015). (Right) Bland‐Altman plots for MRE+PDFF and MRE‐only respectively, each compared to Ristretto MRE (Difference = Proposed − Reference). Limits of agreement (LoA) were SWS: [−0.15, 0.04] m/s, G_l_: [−0.22, 0.12] kPA, |G*|: [−0.39, 0.13] kPA for MRE + PDFF vs. Ristretto MRE, and SWS: [−0.13, 0.15] m/s, G_l_: [−0.21, 0.23] kPa, |G*|: [−0.34, 0.46] kPa for MRE‐only vs. Ristretto MRE.

**FIGURE 6:**
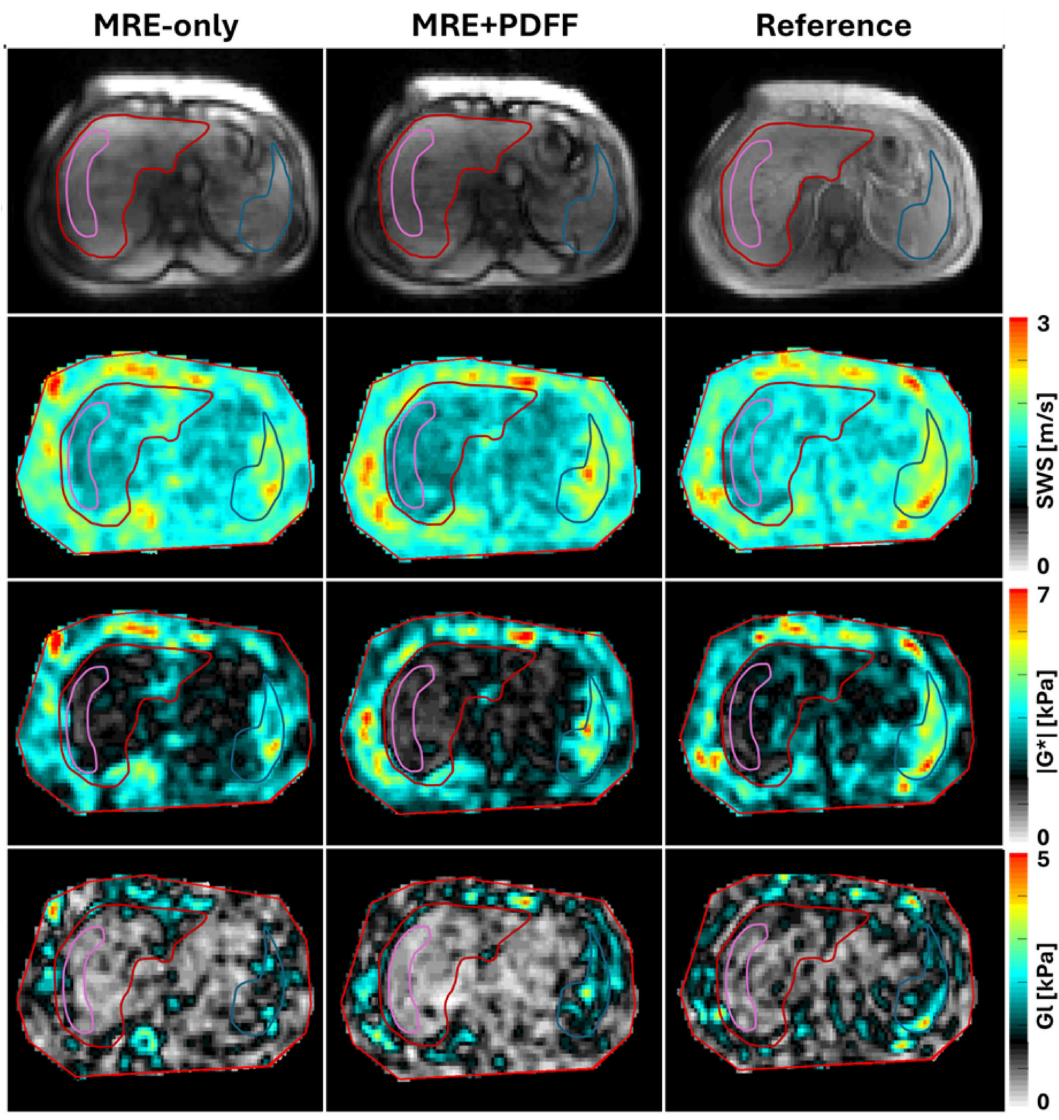
Magnitude images with corresponding shear‐wave speed (SWS, m/s), magnitude of the complex shear modulus (|G*|, kPa), and loss modulus (G_l_, kPa) maps in a representative volunteer for MRE‐only (left), MRE + PDFF (middle), and the four‐breathhold Ristretto MRE scan (right). The whole liver is delineated in red, the liver ROI in pink, and the spleen is shown in blue.

All eight volunteers (one female, 29.4 ± 3.7 years, BMI 23.8 ± 4.8 kg/m^2^) successfully completed the MRI acquisitions. The average reconstruction time for the offline implementation of the algorithm described in Section 2.2.1 was 110 seconds. A comparison of the obtained SWS, |G*|, and G_l_, for MRE‐only, MRE+PDFF, and Ristretto MRE, is shown in Figure 5. Average SWS values were 1.43 ± 0.17 m/s, 1.36 ± 0.15 m/s, and 1.42 ± 0.11 m/s for the MRE‐only, MRE + PDFF, and Ristretto MRE, respectively. Comparison between each acquisition revealed a significant decrease (*p* = 0.015) in SWS for MRE + PDFF compared to Ristretto MRE. Average |G*| values were 1.97 ± 0.48 kPa, 1.78 ± 0.41 kPa, and 1.91 ± 0.30 kPa for MRE‐only, MRE + PDFF, and Ristretto MRE, respectively. The average liver loss modulus was 0.76 ± 0.18 kPa, 0.70 ± 0.16 kPa, and 0.75 ± 0.11 kPa for MRE‐only, MRE + PDFF, and Ristretto MRE, respectively. There were no statistically significant differences between the different acquisition strategies in the magnitude of the complex shear modulus or in the loss modulus. Examples of viscoelastic maps for all acquisition strategies in a representative volunteer can be found in Figure 6. Shear‐wave‐speed values for the kidneys and spleen are provided in Supporting Information Table S2a, and MRE results from the in vivo repeatability study can be found in Supporting Information Table S3a.

#### 3.2.2 PDFF and R_2_*

**FIGURE 7:**
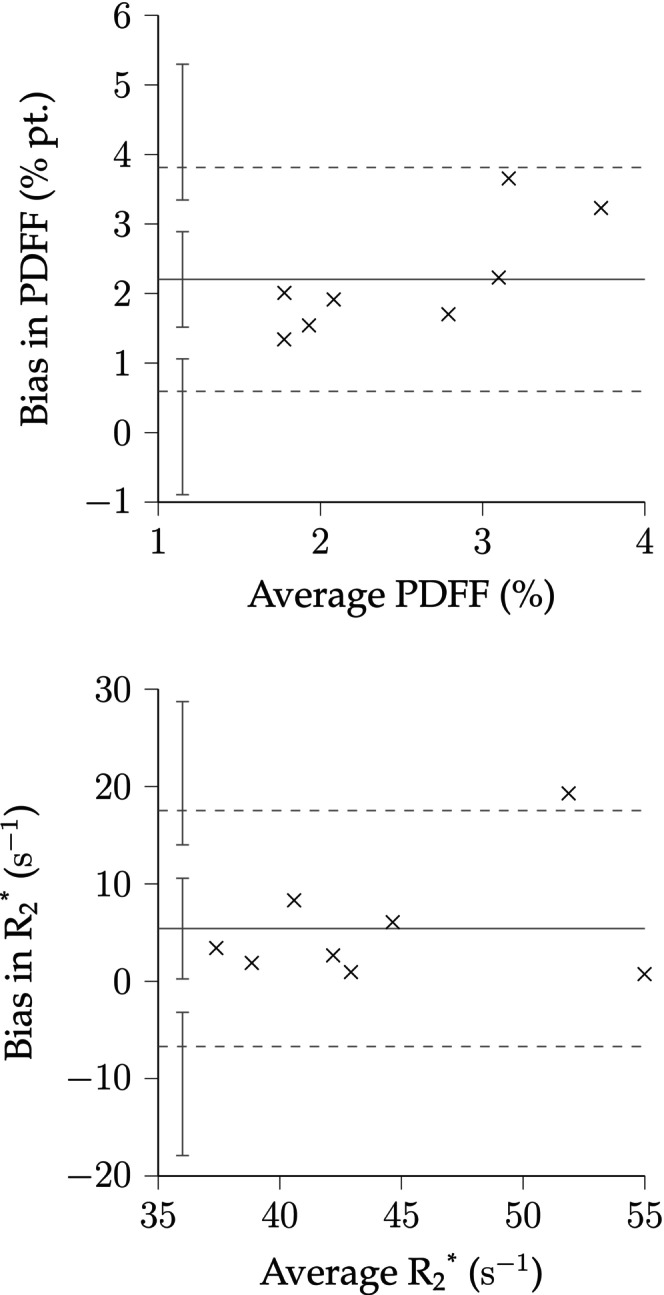
Average liver PDFF (top) and R_2_* (bottom) values for all healthy subjects, for the proposed MRE+PDFF sequence compared with the reference qDixon VIBE acquisition. Solid lines denote mean bias, dashed lines give limits of agreement (± 1.96 SD) and error bars give 95% confidence intervals of the corresponding statistic.

**FIGURE 8:**
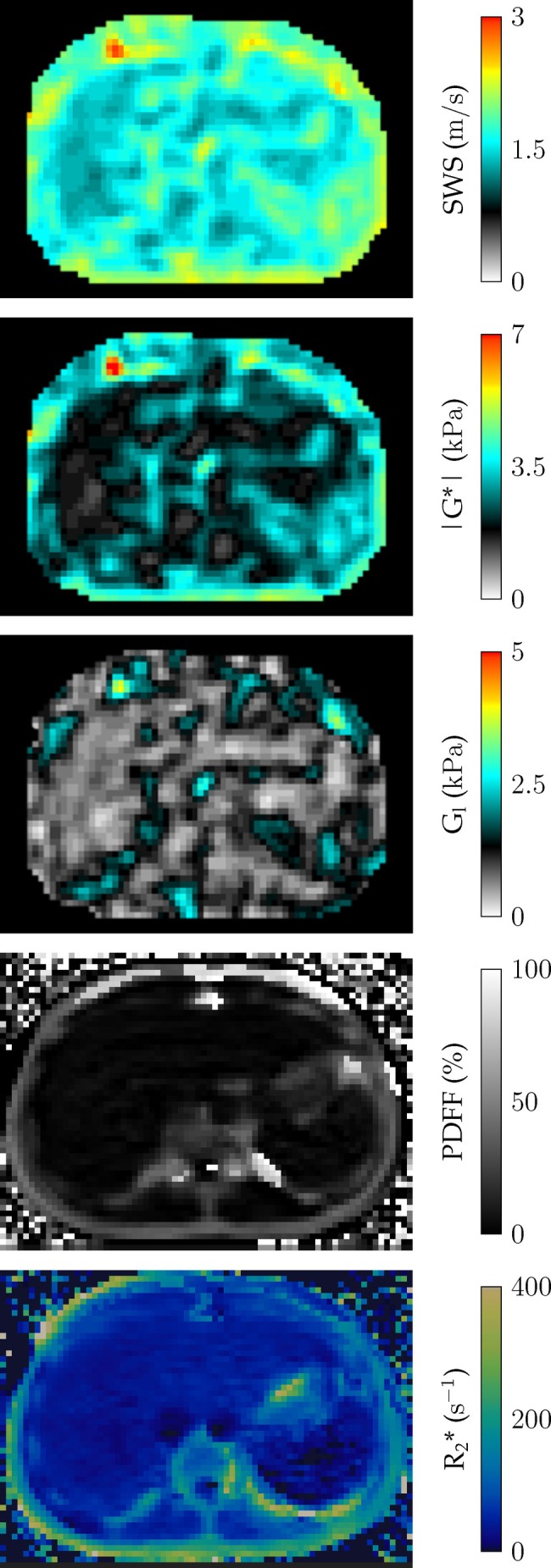
Coregistered viscoelastic, PDFF, and R_2_* parameter maps acquired simultaneously with the proposed sequence.

**FIGURE 9:**
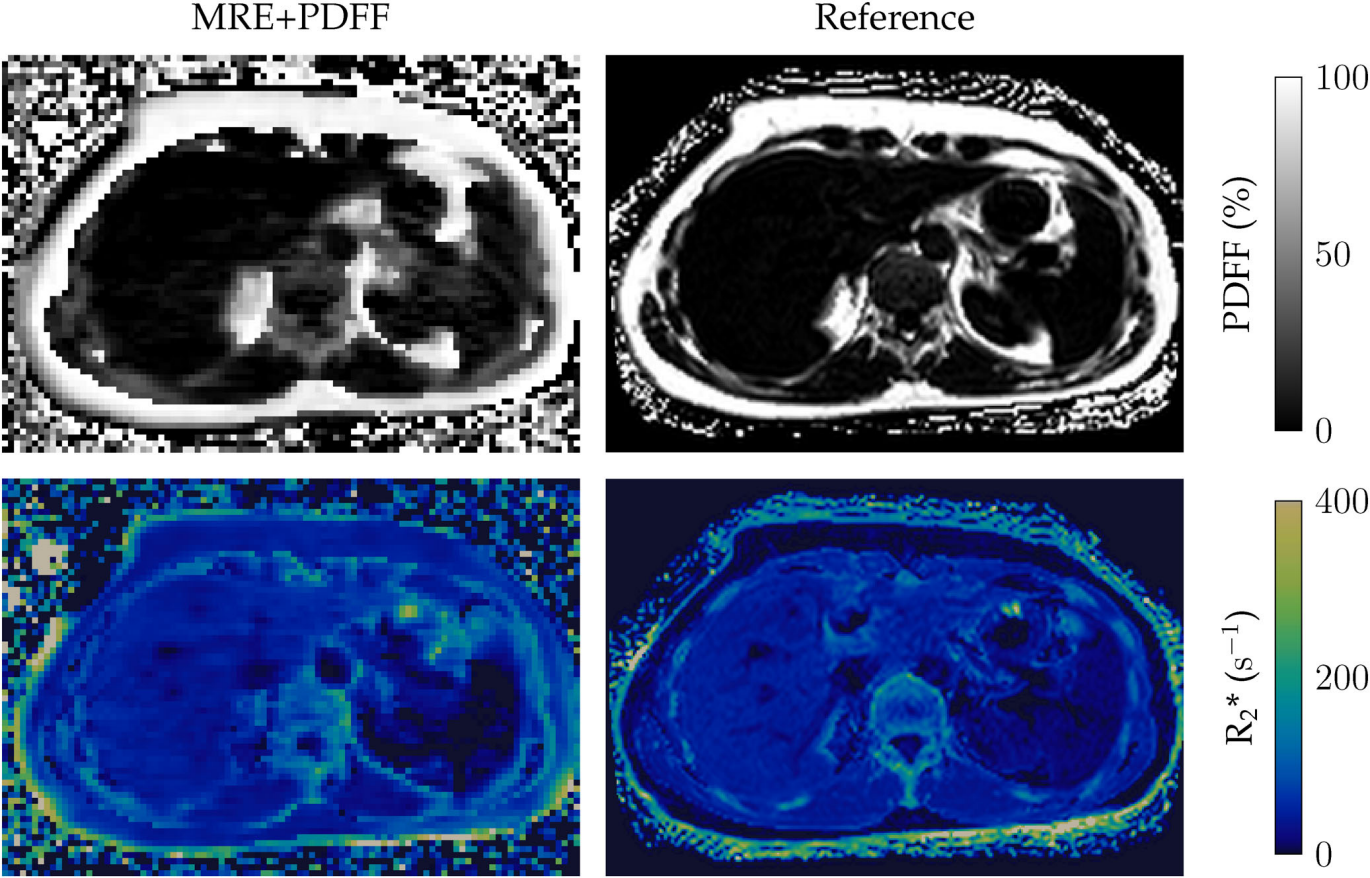
PDFF and R_2_* maps from the proposed MRE+PDFF sequence in a representative volunteer, compared with corresponding scans from the reference qDixon VIBE acquisition.

The mean liver PDFF across all subjects was 3.6% ± 1.0% for MRE+PDFF and 1.4% ± 0.5% for the reference acquisition, whereas mean liver R_2_* values were 46.9 ± 7.3 s^−1^ and 41.5 ± 5.6 s^−1^ for MRE+PDFF and the reference acquisition respectively. These values are compared for all subjects in Figure 7 using Bland–Altman plots. There was a statistically significant bias in liver PDFF values from the proposed MRE+PDFF technique when compared to reference measurements of 2.2 percentage points (*p* < 10^−3^), and the bias in liver R_2_* measurements of 5.4 s^−1^ was also significant (*p* < 0.05). 95% limits of agreement were {0.6, 3.8} % and {−6.76.7, 17.5} s^−1^ for liver PDFF and R_2_* respectively. Example co‐registered viscoelastic, PDFF, and R_2_* maps acquired in a healthy subject can be found in Figure 8, and Figure 9 compares PDFF and R_2_* maps from another healthy subject to corresponding maps from the qDixon VIBE reference acquisition. R_2_* values in the kidneys and spleen are provided in Supporting Information Table S2b, and PDFF and R_2_* results from the in vivo repeatability study can be found in Supporting Information Table S3b.

## 4 Discussion

This work proposes a 3D slab‐selective MRE acquisition, with the additional possibility of simultaneous PDFF and R_2_* measurement. The proposed sequence uses a highly time‐efficient interleaved motion‐encoding scheme, together with incoherent k‐space undersampling, to permit the acquisition of complete 3D MRE data in a single breathhold. Viscoelastic maps are computed following a model‐regularised reconstruction, while individual echoes for PDFF and R_2_* estimation can be reconstructed directly from a combination of k‐space samples from the reference scan acquired across different wave offsets. The isotropic 4 mm resolution and 32 mm axial field‐of‐view achieved are comparable to existing techniques for 3D liver MRE [26]. The PDFF and R_2_* maps produced are guaranteed to be co‐registered, both with each other and with all viscoelastic maps, as all measured parameters are acquired in the same breathhold.

Good quantitative agreement between the proposed sequence and established methods was observed both in a range of phantoms and in a small cohort of healthy subjects. For viscoelastic parameters, a statistically significant bias was only observed between the shear‐wave speeds obtained in vivo with the proposed MRE+PDFF and the Ristretto MRE reference. This bias of −0.06 m/s is small compared to the range of values encountered in patients, where a difference of approximately 0.2 m/s is observed between consecutive fibrosis scores [24]. Wider limits of agreement were observed for the MRE‐only sequence compared to the MRE+PDFF sequence, and this may be due to the use of higher acceleration for the MRE‐only sequence, as observed in a previous quantitative liver MRI study [59].

Repeatability of MRE measurements with the proposed technique was similar to or better than Ristretto in all cases, with the exception of G_l_ measurements with the MRE‐only sequence in the gelatin phantom, which had a slightly increased coefficient of variation across measurements compared to Ristretto (17% versus 14%). As discussed above, the higher acceleration of the MRE‐only sequence may explain this increased variability, and this is especially true for measurements of G_l_, which are sensitive to noise [49]. The geometry of the gelatin phantom also led to the presence of standing waves, which make accurate estimation of G_l_ challenging.

In vivo measurements of PDFF and R_2_* both exhibited small but statistically significant biases compared to reference measurements. However, the mean PDFF observed with the MRE+PDFF sequence of 3.6% ± 1.0% is below the diagnostic threshhold of 5 or 6% [60, 61]. Nevertheless, it is worth considering potential sources for this bias. Of the effects known to confound PDFF measurement [62], the proposed technique accounts for R_2_* and the multi‐peak spectrum of fat, but does not correct for T_1_ bias. The flip angle of the MRE+PDFF sequence in vivo was set to be close to the Ernst angle for a liver measurement at 3 T (about 9 degrees for a T_R_ of 9.38 ms, assuming a liver T_1_ of 800 ms [63]). The influence of T_1_ could likely be reduced through the choice of a lower flip angle, as was done for the phantom experiments (where no systematic bias in PDFF measurements was observed), but this may lead to a reduced SNR. Another difference between the phantom and in vivo experiments was in the methods used for parameter estimation: whereas for the phantom experiments all PDFF and R_2_* values were computed offline, the in vivo reference values were provided by a proprietary vendor implementation, which may have introduced a bias between our technique and the reference. This could be confirmed by applying the vendor implementation to the data from the proposed sequence, but this was not possible in this study due to the lack of an inline implementation of the proposed reconstruction. Other possible sources of bias include aliasing of the subcutaneous fat into the liver. This could occur in the form of either residual undersampling artefacts or respiratory motion artefacts, with the latter being more probable, as some subjects struggled to maintain a breathhold while the mechanical vibration was active.

Compared to other recently proposed techniques for single‐breathhold 3D abdominal elastography which use wave offset interleaving [27], the interleaved‐motion‐encoding scheme proposed here achieves greater time efficiency because it enables a shorter repetition time. Further, the proposed framework utilises a sufficiently low degree of undersampling that MRE image reconstruction can be performed without spatial regularisation. Instead, a model‐based temporal regularisation is used for reconstruction of the motion‐encoded images, along similar lines to the work of Mohammed et al. [64], where a model fit relating the displacement phasor to the phase images is performed as part of the image reconstruction. In that work, this is done so that spatial regularisation can be applied to the displacement phasor, whereas in this work the result of the model fit itself is used to regularise the image reconstruction, and no spatial regularisation is performed. Compared to 2D multislice techniques, including accelerated simultaneous multislice techniques [26, 65], a 3D volumetric acquisition offers increased signal‐to‐noise ratio. Further, simultaneous multislice techniques may suffer from additional loss of SNR due to high acceleration factors along the slice direction paired with poor *g* factors due to limited spatial coverage in the slice direction, and hence a lack of receiver coil profile variation [26].

The proposed approach also offers advantages over other accelerated MRE techniques. First of all, it does not suffer from the geometric distortions of EPI‐based techniques [66]. Geometric fidelity is particularly relevant in MRE because MRE inversion requires spatial derivatives and therefore relies on positional accuracy of the acquired data. It should also be noted that it is not straightforward to incorporate PDFF and R_2_* mapping into EPI‐based techniques for two reasons: firstly because the EPI readout already requires rapid switching of the readout gradient, and secondly because the EPI MRE techniques used clinically acquire spin echoes [67], which are sensitive to R_2_ rather than R_2_*. Spiral trajectories [68, 69] have also been exploited in MRE for their scanning efficiency, and have been used to enable dynamic monitoring of liver stiffness with high temporal resolution [70]. However, compared to the Cartesian k‐space trajectory of the proposed acquisition, spiral trajectories are more susceptible to off‐resonance effects and eddy currents, especially in the presence of strong motion‐encoding gradients. A recently proposed technique uses a stack‐of‐stars radial trajectory with a DENSE multi‐slab acquisition and Hadamard encoding for fast brain MRE [71], but its use of stimulated echoes incurs a 50% SNR penalty. Another recent work proposed distributed motion‐encoding schemes to accelerate brain MRE scans [72]. In this work, the traditional MRE encoding scheme was retained in order to exploit the reference scan for PDFF and R_2_* mapping. Nevertheless, it would be possible to extend the proposed MRE‐only approach by pairing an incoherent distributed encoding across wave offsets with a joint reconstruction of all motion‐encoding directions, and this might enable higher acceleration factors.

Another approach to address breathhold limitations is to enable a free‐breathing acquisition. For example, radial free‐breathing MRE has demonstrated strong agreement with conventional Cartesian breath‐hold methods in hepatic stiffness measurements [73]. However, care must be taken with radial free‐breathing simultaneous R_2_* mapping as without proper respiratory gating or motion correction, free‐breathing approaches risk overestimating R_2_* values due to residual motion artifacts [74, 75, 76]. Moreover, free‐breathing liver MRE techniques do not allow the acquisition to be performed during a mechanical steady state.

The implementation of the multi‐echo acquisition for PDFF and R_2_* quantification, the readout gradients of which are superimposed on the motion‐encoding gradients, required a reduction in the MRE motion encoding gradient amplitude to avoid exceeding maximum available gradient slew rate on the scanner. The six‐lobe readout gradient waveform was included in the motion‐encoded acquisitions as ideally the only difference between these and the reference acquisition should be the presence of the motion‐encoding gradients. This ensures that the reference scan can provide the correct background phase information (including the effects of eddy currents). This background phase is also a confounding factor for PDFF and R_2_* estimation, which was addressed in this work using the magnitude‐based fitting approach proposed by Zhong et al. [35]. The acceleration factor was also reduced for acquisitions including simultaneous PDFF and R_2_* mapping, as a degradation in the point spread function (even minor undersampling artefacts) can compromise PDFF quantification accuracy by introducing aliases of the subcutaneous fat signal into the hepatic region. This necessitated an increased–though still clinically feasible–breathhold duration for these acquisitions. While the choice was made not to use spatial regularisation in this work, the addition of spatial regularisation to the reconstruction of the volumes used for PDFF and R_2_* quantification, for example on the total variation or the coefficients of the wavelet transform [77], would be a straightforward way to achieve higher accelerations, and hence shorter acquisition times, if required. The implementation of the proposed reconstruction used a fixed iteration count, which had to be manually reduced to prevent noise amplification in the reconstruction of the gelatin phantom. This was because the reconstruction of the small, featureless phantom converged much more quickly than in the healthy volunteers. A suitable early‐stopping criterion would prevent the need for this manual tuning, and also offer potential savings in reconstruction time.

Combining MRE with simultaneous PDFF and R2* mapping offers a comprehensive, non‐invasive approach to evaluating chronic liver disease. It has been shown that the combination of MRE and PDFF improves diagnostic specificity for diagnosing nonalcoholic steatohepatitis [78]. R_2_* mapping adds value by detecting concurrent iron overload, which can confound stiffness measurements or indicate secondary etiologies like hereditary hemochromatosis [79]. Combining the insights of these modalities allows for a more nuanced understanding of complex liver diseases. While acquiring these scans in series is clinically feasible, it increases the burden on patients, typically requiring around five breathholds: one approximately 20‐s breathhold for 3D CSE‐MRI, and four separate breathholds for 3D MRE (three motion‐encoded acquisitions and a reference scan). In contrast, the proposed approach enables acquisition in a single breathhold, also producing co‐registered parametric maps. Previous work has shown initial results of a 2D MRE sequence using a two‐point Dixon readout for hepatic liver stiffness and fat quantification [80]. By comparison, the proposed sequence has a 3D slab‐selective acquisition, utilising 3D MRE for viscoelastic‐parameter mapping, and additionally includes R_2_* mapping, which allows for R_2_*‐corrected PDFF estimation. A limitation of the proposed technique is its reduced spatial coverage when compared to full‐liver PDFF and R_2_* scans [81]. However, there are existing MR techniques with limited spatial coverage that see clinical usage for fat and iron quantification, such as single‐voxel MR spectroscopy [82]. Another limitation is that the proposed technique is not effective in the presence of very high R_2_* values due to the rapid signal decay across the acquired echoes, but this is a limitation shared by similar CSE‐MRI techniques, where R_2_* values greater than 779 s^−1^ have been shown to prevent reliable PDFF estimation at 3 T [83]. This motivated the exclusion of the vials with R_2_* values of 900 s^−1^ and 1500 s^−1^ from the analysis of the PDFF/R_2_* phantom experiments. Such high R_2_* values can be encountered in the liver in vivo, but this is rare [84].

This study was limited in the scope of the subjects for in vivo validation. Notably, the proposed sequence was only tested in a limited number of healthy volunteers, with no liver fat or iron deposition, and normal viscoelastic values. Therefore, the in vivo results cannot be generalized to the full range of PDFF, R_2_* and MRE values, and further validation in patients with a wider range of PDFF, R_2_*, stiffness and viscosity values is required. The average BMI of the healthy volunteer cohort was only 23.8, whereas for MASLD patients this is likely to be higher. Imaging can be more challenging in higher BMI subjects due to the increase in distance between coil arrays and the centre of the imaged volume, leading to decreased coil sensitivity. Imaging was also only performed in healthy subjects, so the diagnostic capability of the proposed sequence is yet to be proven. The proposed sequence was only demonstrated at a single field strength, but should be transferable to other clinically relevant field strengths, including 0.55 T where MRE has been shown to be feasible [85] and could benefit from shorter scan times. Further studies are therefore required, incorporating additional field strengths, known MASLD patients, and high‐BMI subjects.

## 5 Conclusions

We have proposed a fast, 3D slab‐selective MRE sequence, and shown that it can be combined with PDFF and R_2_* mapping in a single‐breathhold liver scan. Testing in phantoms and healthy subjects demonstrated good quantitative accuracy and in vivo feasibility. A study in MASLD patients is warranted to evaluate the robustness and clinical benefit of the proposed approach.

## Funding

This work was supported by the EPSRC Centre for Doctoral Training in Smart Medical Imaging (Grant No. EP/S022104/1), British Heart Foundation (Grant Nos. PG/18/59/33955, RE/18/2/34213, RG/20/1/34802), Wellcome EPSRC Centre for Medical Engineering (Grant No. NS/A000049/1), Siemens Healthineers, Engineering and Physical Sciences Research Council (Grant Nos. EP/L015226, EP/P001009/1, EP/P007619, EP/P032311/1, EP/V044087/1), Center of Interventional Medicine for Precision and Advanced Cellular Therapy (#FB210024), National Institute for Health and Care Research, Millennium Institute for Intelligent Healthcare Engineering (Grant No. ICN2021_004), Fondo Nacional de Desarrollo Científico y Tecnológico (Grant Nos. 1250252, 1250261), and Technische Universität München.

## Disclosure

This research was supported in part by the Intramural Research Program of the National Institute of Diabetes and Digestive and Kidney Diseases (NIDDK) within the National Institutes of Health (NIH). The contributions of the NIH author(s) are considered Works of the United States Government. The findings and conclusions presented in this paper are those of the author(s) and do not necessarily reflect the views of the NIH or the U.S. Department of Health and Human Services.

## Conflicts of Interest

Dr. Omar Darwish and Dr. Karl P Kunze are employees of Siemens Healthineers. The other authors declare no conflicts of interest.

## Supporting information




**Table S1:** Coefficients of variation for viscoelastic parameters in the ultrasound gel (a) and gelatin (b) phantoms from the repeatability study.
**Figure S1:** Linear regression analysis of PDFF (left) and R_2_* (right) measurements with MRE+PDFF (a) and qDixon VIBE (b) acquisitions.
**Figure S2:** Coefficients of variation of PDFF (left) and R_2_* (right) measurements in the PDFF/R_2_* phantom from the repeatability study. The vial with PDFF = 0% is omitted; in this vial, the standard deviation across repetitions was 0.12% pt. for MRE+PDFF, and for qDixon VIBE this was 0.05% pt.
**Table S2:** Mean shear‐wave speed (a) and R_2_* (b) values in the left and right kidneys and the spleen.
**Table S3:** Intraclass correlation coefficients for mean liver viscoelastic parameters (a) and PDFF and R_2_* (b) from the repeatability study in healthy volunteers.

## Data Availability

The data that support the findings of this study are available from the corresponding author upon reasonable request.
