# Supplementary material for: Single‐Breathhold 3D MR Elastography in the Liver, With Simultaneous R2* and PDFF Mapping
Source: Magn Reson Med. 2026 Apr 19;96(2):682–97. doi: 10.1002/mrm.70372 (PMC13269237; doi:10.1002/mrm.70372)
Supplement: Supplementary file 1 — Table S1: Coefficients of variation for viscoelastic parameters in the ultrasound gel (a) and gelatin (b) phantoms from the repeatability study. Figure S1: Linear regression analysis of PDFF (left) and R2* (right) measurements with MRE+PDFF (a) and qDixon VIBE (b) acquisitions. Figure S2: Coefficients of variation of PDFF (left) and R2* (right) measurements in the PDFF/R2* phantom from the repeatability study. The vial with PDFF = 0% is omitted; in this vial, the standard deviation across repetitions was 0.12% pt. for MRE+PDFF, and for qDixon VIBE this was 0.05% pt. Table S2: Mean shear‐wave speed (a) and R2* (b) values in the left and right kidneys and the spleen. Table S3: Intraclass correlation coefficients for mean liver viscoelastic parameters (a) and PDFF and R2* (b) from the repeatability study in healthy volunteers. [file MRM-96-682-s001.pdf]

Supporting Information

Table S1: Coefficients of variation for viscoelastic parameters in the ultrasound gel (a) and gelatin (b) phantoms from the repeatability study.

(a) Ultrasound gel

	MRE-only	MRE+PDFF	Ristretto
c_s	0.7%	1.1%	1.3%
$\|G^*\|$	1.4%	2.0%	3.5%
G_l	7.6%	8.8%	5.2%

(b) Gelatin

	MRE-only	MRE+PDFF	Ristretto
c_s	3.6%	2.6%	2.9%
$\|G^*\|$	6.6%	5.4%	5.9%
G_l	17.2%	12.4%	13.9%

(a) MRE+PDFF

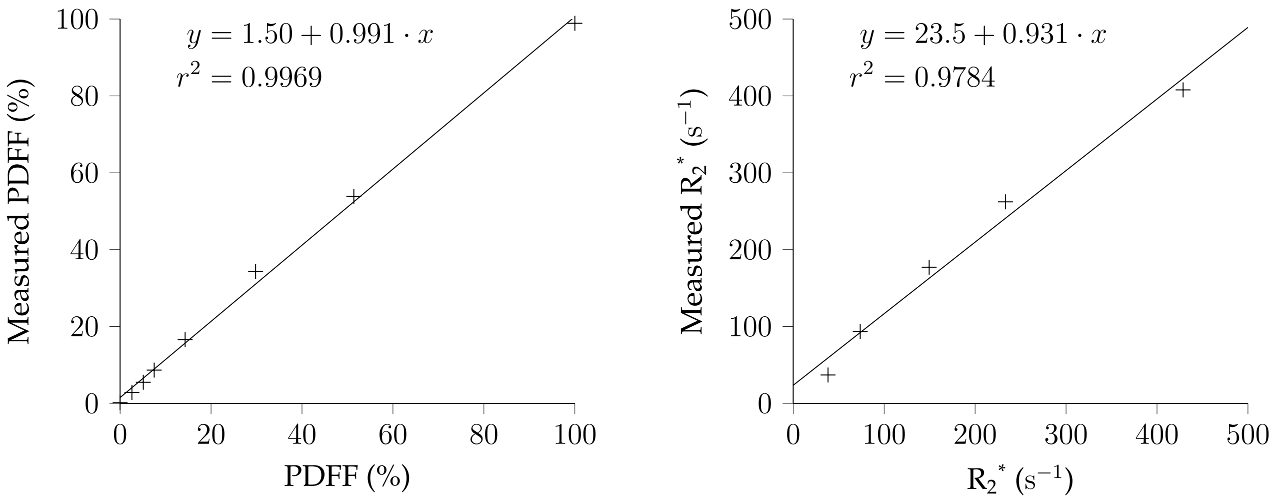

(b) qDixon VIBE

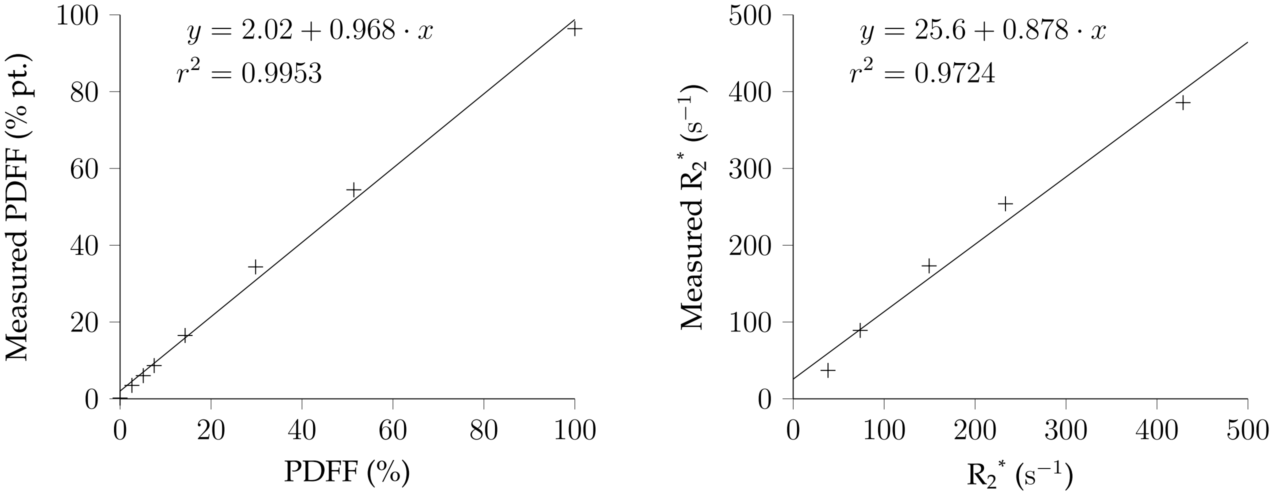

Figure S1: Linear regression analysis of PDFF (left) and R_{2*} (right) measurements with MRE+PDFF (a) and qDixon VIBE (b) acquisitions.

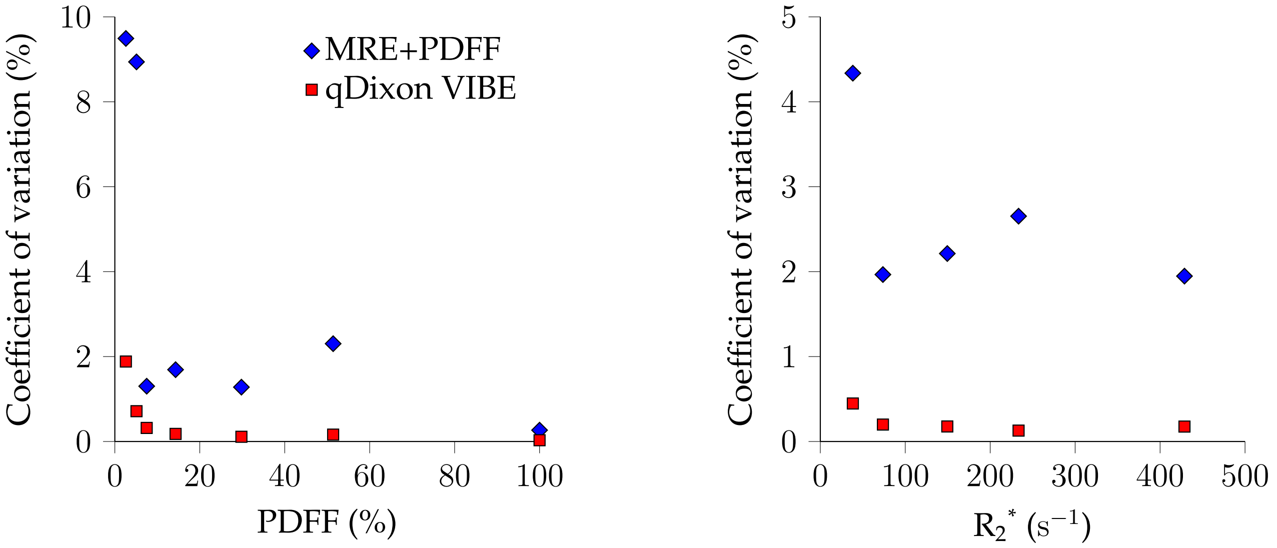

Figure S2: Coefficients of variation of PDFF (left) and R_2* (right) measurements in the PDFF/R_2* phantom from the repeatability study. The vial with PDFF = 0% is omitted; in this vial, the standard deviation across repetitions was 0.12% pt. for MRE+PDFF, and for qDixon VIBE this was 0.05% pt.

Table S2: Mean shear-wave speed (a) and R_2* (b) values in the left and right kidneys and the spleen.

(a) c_s (m/s)

	MRE-only	MRE+PDFF	Ristretto
Left kidney	1.77	1.68	1.73
Right kidney	1.66	1.66	1.71
Spleen	1.95	2.01	2.05

(b) R_2* (s^{-1})

	MRE+PDFF	qDixon VIBE
Left kidney	23.3	17.7
Right kidney	20.6	16.3
Spleen	19.1	20.3

Table S3: Intraclass correlation coefficients for mean liver viscoelastic parameters (a) and PDFF and R_2* (b) from the repeatability study in healthy volunteers.

(a) Viscoelastic parameters

	MRE-only	MRE+PDFF	Ristretto
c_s	0.969	0.907	0.909
$\|G^*\|$	0.984	0.931	0.899
G_l	0.857	0.914	0.753

(b) PDFF and R_2*

	MRE+PDFF	qDixon VIBE
PDFF (%)	0.907	0.929
R_2* (s^{-1})	0.906	0.913
